# The Incidence, Mortality and Medical Expenditure in Patients with Asthma in Taiwan: Ten-year Nationwide Study

**DOI:** 10.1007/s44197-024-00230-8

**Published:** 2024-04-24

**Authors:** Kuang-Ming Liao, Pei-Jun Chen, Yu-Tung Hung, Tzu-Ju Hsu, Fuu-Jen Tsai, Te-Chun Shen

**Affiliations:** 1 Department of Internal Medicine, Chi Mei Medical Center, Chiali, Tainan, Taiwan; 2 Department of Nursing, Min-Hwei Junior College of Health Care Management, Tainan, Taiwan; 3 Department of Nursing, Nantou Hospital, Ministry of Health and Welfare, Nantou, Taiwan; 4 Clinical Trial Center, Management Office for Health Data, China Medical University Hospital, Taichung, Taiwan; 5 Division of Pulmonary and Critical Care Medicine, Department of Internal Medicine, China Medical University Hospital, No. 2 Yude Road, 404 Taichung, Taiwan; 6 School of Medicine, China Medical University, Taichung, Taiwan; 7 Division of Critical Care Medicine, Chu Shang Show Chwan Hospital, Nantou, Taiwan

**Keywords:** Asthma, Incidence, Mortality, Medical Expenditure

## Abstract

**Background:**

This study examines incidence, mortality, medical expenditure and prescription patterns for asthma on a national scale, particularly in Asian countries for asthma is limited. Our aim is to investigate incidence, mortality, prescription patterns and provide a comprehensive overview of healthcare utilization trends for asthma from 2009 to 2018.

**Methods:**

We included patients diagnosed with asthma between 2009 and 2018. We excluded patients with missing demographic data. Our analysis covered comorbidities, including diabetes mellitus, hypertension, allergic rhinitis, eczema, atopic dermatitis, coronary artery disease, congestive heart failure, chronic kidney disease, chronic hepatitis, stroke, and cancer. Investigated medications comprised oral and intravenous steroids, short-acting beta-agonists, inhaled corticosteroids (ICS), combinations of ICS and long-acting beta-agonists, long-acting muscarinic antagonists, and leukotriene receptor antagonists montelukast. We also assessed the number of outpatient visits, emergency visits, and hospitalizations per year, as well as the average length of hospitalization and average medical costs.

**Results:**

The study included a final count of 88,244 subjects from 1,998,311 randomly selected samples between 2000 and 2019. Over the past decade, there was a gradual decline in newly diagnosed asthma patients per year, from 10,140 to 6,487. The mean age annually increased from 47.59 in 2009 to 53.41 in 2018. Over 55% of the patients were female. Eczema was diagnosed in over 55% of the patients. Around 90% of the patients used oral steroids, with a peak of 97.29% in 2018, while the usage of ICS varied between 86.20% and 91.75%. Intravenous steroids use rose from 40.94% in 2009 to 54.14% in 2018. The average annual hospital stay ranged from 9 to 12 days, with a maximum of 12.26 days in 2013. Lastly, the average medical expenses per year ranged from New Taiwan dollars 5558 to 7921.

**Conclusions:**

In summary, both asthma incidence and all-cause mortality rates decreased in Taiwan from 2009 to 2018. Further analysis of medical expenses in patients with asthma who required multiple hospitalizations annually revealed an increase in outpatient and emergency visits and hospitalizations, along with longer hospital stays and higher medical costs.

## Introduction

Asthma is a widespread, long-term lung condition that impacts numerous individuals across various nations globally [1]. It is marked by fluctuating signs such as coughing, wheezing, shortness of breath, and chest constriction, alongside inconsistent limitations in the outflow of breath. These symptoms and breathing restrictions typically fluctuate in their severity and occurrence over time. Over the last ten years, advancements have been achieved in asthma management, especially regarding creative strategies that utilize current medications and new treatments for severe cases of asthma. Previously, for mild asthma, guidelines recommended a regimen of long-term low-dose inhaled corticosteroids (ICS) and as-needed short-acting beta-agonists (SABA) [2]. However, many patients with mild asthma show poor adherence to daily ICS due to sporadic symptoms, leading to a heavy reliance on SABA during acute exacerbation. Despite their seemingly mild condition, these patients remain at risk for serious asthma attacks and related fatalities. Additionally, both exclusive SABA treatment and its excessive use are linked to negative disease outcomes. These issues have prompted the asthma care community to explore new strategies and reconsider the existing approach to enhance the management of asthma [3]. A new once-daily fluticasone furoate (FF) was a monotherapy therapy for asthma, and when combined with vilanterol (VI), a novel once-daily inhaled long-acting beta-agonists (LABA), it is being studied for both asthma management and significant airway protection in individuals with asthma [4]. The Salford Lung Study [5] is a comprehensive Phase III trial involving over 4,000 patients across multiple centers. This open-label, randomized controlled trial aims to assess the efficacy and safety of starting treatment with FF/VI to the standard care therapy for asthma over 52 weeks. Patients who began treatment with FF/VI demonstrated better improvements in asthma control compared to those who continued with their regular treatment, which typically includes ICS either alone or in combination with LABA. Tiotropium has proven beneficial as an additional maintenance therapy alongside medium or high-dose ICS, with or without LABA, for asthma treatment [6]. It has gained approval in the USA, countries in Europe for this use. Studies in adults have shown that tiotropium improved lung function and asthma management while safety profile was similar to placebo [7]. Moreover, clinical trials in children with symptomatic asthma despite received ICS therapy indicate that tiotropium, when used as a supplementary treatment to ICS is safe and effective. It also shows a tendency to improve asthma control, echoing the results observed in adult populations [8]. While the development of innovative relief strategies and treatments for severe asthma marks an exciting advancement, numerous challenges remain. Current clinical trials are in progress, aiming to address and overcome some of these challenges. The International Study of Asthma and Allergies in Childhood (ISAAC) is a standardized asthma survey since 1995 [9]. Global Asthma Network (GAN), began in 2012 [10], after the ISAAC and observed that the prevalence of asthma symptoms was on the rise in several countries, and some disease severities were high. GAN Phase I extends the research of ISAAC by gathering additional data on the prevalence, severity, diagnosis, and management of asthma, along with some allergic disease. It also looks into emergency room visits, hospital admissions, and the usage of essential asthma medications. Taiwan participated in this study, concentrating on a demographic of adolescents aged 13–14 years who completed a questionnaire in 2017. The objective was to explore the current prevalence of asthma in Taiwanese teens. However, the study was limited to only surveying individuals within the 13-14-year age group [11]. The aim of our study utilized nationwide claims data to analyze the yearly patterns of asthma in Taiwan over the period from 2009 to 2018. Additionally, it assessed the trends in the incidence and mortality rates of patients with asthma and examined the medical expenditure, identifying the shifts in these aspects among asthma patients in Taiwan throughout the decade.

## Materials and methods

### Data Source

On January 1, 1995, the Department of Health of the Executive Yuan formally established the Central Health Insurance Bureau, and on March 1, 1995, the National Health Insurance was officially implemented. So far, the national acceptance rate is as high as 99%. Since 2000, it has provided value-added services to the academic health insurance database on the premise of protecting public privacy and data security to facilitate public health and academic research. The disease coding for National Health Insurance (NHI) database follows the International Classification of Diseases, 9th Revision and 10th Revision, Clinical Modification (ICD-9-CM and ICD-10-CM). This study was approved by the Institutional Review Board of the Research Ethics Committee of China Medical University Hospital (CMUH110-REC1-038(CR-2)).

### Study Population and Variables

**Fig. 1:**
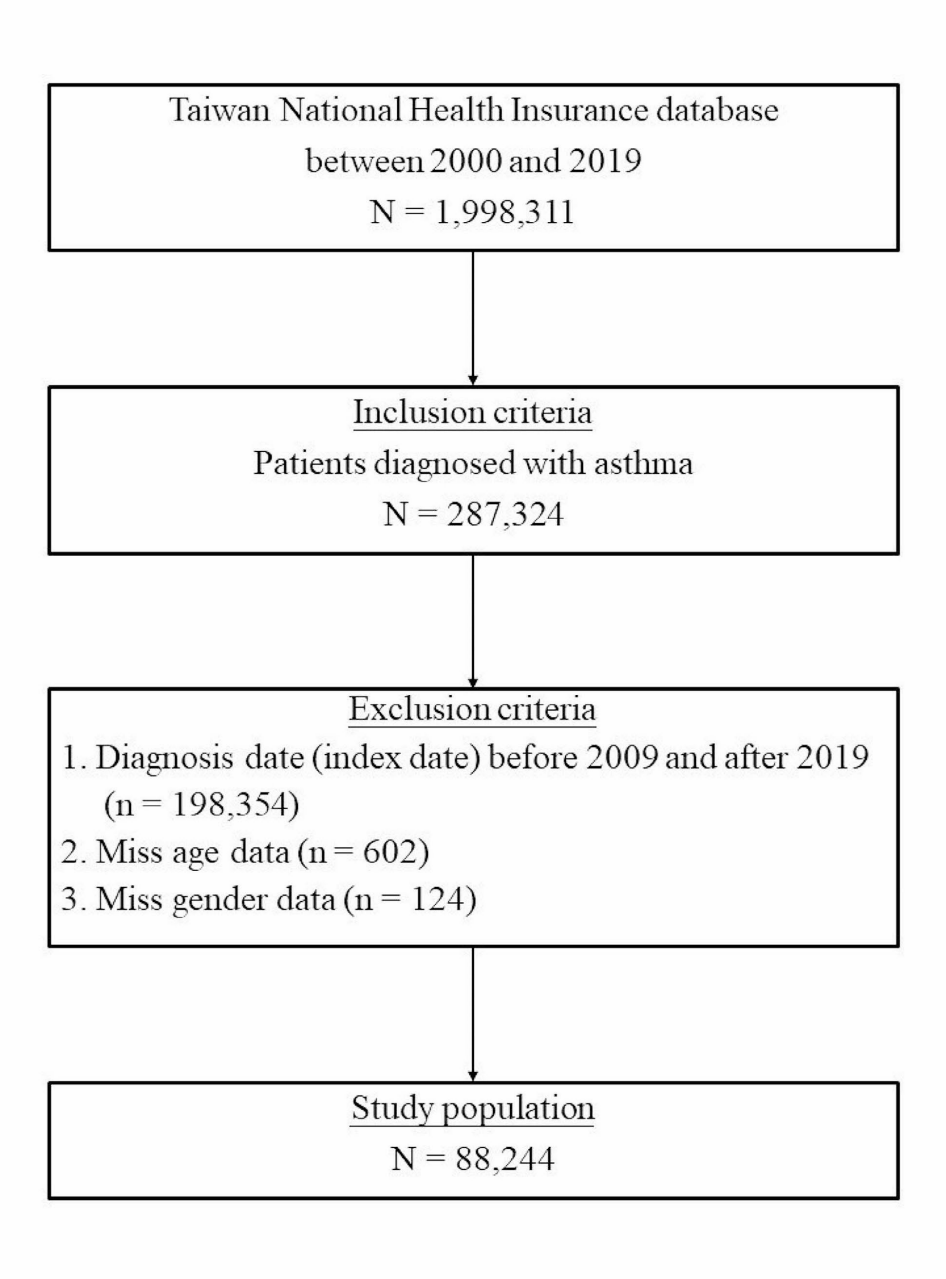
The flow chart of study sample selection from NHI database in Taiwan

Asthma (ICD-9-CM: 493; ICD-10-CM: J45) patients with at least two outpatient visits or one hospitalization record were identified between January 1, 2009 and December 31, 2018; the date of the first record was defined as the date of asthma diagnosis (index date). The exclusion criteria include those missing age or gender data. Figure 1 was the flow chart of study design. The final number of subjects included was 88,244.

The variables in the study were age group (< 20, 20–29, 30–39, 40–49, 50–59, ≥ 60), gender, comorbidities [diabetes mellitus (ICD-9-CM: 250; ICD-10-CM: E08-E13), hypertension (ICD-9-CM: 401–405; ICD-10-CM: I10-I15), allergic rhinitis (ICD-9-CM: 477; ICD-10-CM: J30), eczema (ICD-9-CM: 692.0-692.6, 692.81, 692.83, 692.89, 692.9; ICD-10-CM: L23-L25, L30.0, L30.2, L30.8, L30.9), atopic dermatitis (ICD-9-CM: 691; ICD-10-CM: L20), dyslipidemia (ICD-9-CM 272; ICD-10-CM: E77, E78), coronary artery disease (ICD-9-CM: 410–414; ICD-10-CM: I20-I25), congestive heart failure (ICD-9-CM: 428.0; ICD-10-CM: I50.2-I50.9), chronic kidney disease (ICD-9-CM: 585; ICD-10-CM: N18.4-N18.9), chronic hepatitis (ICD-9-CM: 571.4; ICD-10-CM: K73), stroke (ICD-9-CM: 430–438; ICD-10-CM: I60-I69), and cancer (ICD-9-CM: 140–208; ICD-10-CM: C00-C99)], medications [oral steroids (anatomical therapeutic chemical (ATC) code: A01AC01, A01AC91, A07EA06, A14AA02, A14AA03, G01AF04, G01AX05, H02AA02, H02AB01, H02AB02, H02AB04, H02AB05, H02AB06, H02AB08, H02AB10, H02BX, H02BX91, M01BA03, S02CA04, S02CA06), intravenous steroids (ATC code: A14AB01, D11AH05, H02AB, H02AB01, H02AB02, H02AB04, H02AB06, H02AB08, H02AB09, H02BX), SABA (ATC code: R03AC), ICS (ATC code: R03BA), ICS/ LABA (ATC code: R03AK), long acting muscarinic antagonists (LAMA) (ATC code: R03BB), and leukotriene receptor antagonists (LTRA) montelukast (ATC code: R03DC)], outpatient department (OPD) visits per year, emergency room (ER) visits per year, hospital admissions per year, average days of hospitalization per year, and average medical costs of asthma care per year.

Detailed variable settings were described as follows. Comorbidities were identified before the asthma diagnosis, evaluating 5-year intervals before the index date. The medication regimen was identified during the first year since the asthma diagnosis. The OPD visits per year are defined and calculated as the total number of OPD visits during the first year since the asthma diagnosis. ER visits per year are defined and calculated as the total number of ER visits during the first year since the asthma diagnosis. Hospital admissions per year are defined and calculated as the total number of hospitalizations during the first year since the asthma diagnosis. The average days of hospitalization per year are defined and calculated as the total days of hospitalizations during the first year since the asthma diagnosis. The medical costs for the OPD visit, ER visit, and hospitalization were available, and all were recorded with the new Taiwan dollar (NTD) in the database. The average medical costs per year were defined and calculated as the total costs for asthma care (OPD, ER, and hospitalization) during the first year since the asthma diagnosis.

### Statistical Analysis

The demographic characteristics of patients diagnosed with asthma in each year from 2009 to 2018 were analyzed. The patient’s age, gender, comorbidities, medications, OPD visits per year, ER visits per year, hospitalizations per year, length of hospitalization per year, and medical costs per year are estimated. Categorical variables were represented by numbers and percentages, while continuous variables were represented by mean and standard deviation (SD). The all-cause mortality rate of asthma per year is calculated by dividing the total number of all-cause deaths of patients with asthma in a certain year (2009–2018) by the total population (the same mid-year), resulting in a rate per 1000 person-years. The incidence rate of asthma per year is determined by dividing the total number of newly diagnosed asthma in a certain year (2009–2018) by the total population (the same mid-year), resulting in a rate per 1000 person-years. All statistical analysis were performed by SAS software, version 9.4 (SAS Institute Inc., Cary, NC).

## Results

The baseline characteristics of patients diagnosed with asthma from 2009 to 2018 are displayed in Table 1, including age, gender, and comorbidities. Over the past decade, there has been a gradual decline in the number of patients, decreasing from 10,140 to 6487. The mean age annually varied from 47.59 (SD = 21.13) to 53.41 (SD = 17.67) years. Throughout these ten years, over half of the patients were female. More than 55% of the patients were diagnosed with eczema, persisting for the entire decade. The second most common comorbidity was allergic rhinitis.

**Table 1:** The baseline characteristics of patients with asthma by year

	2009		2010		2011		2012		2013		2014		2015		2016		2017		2018		
Variables	n	%	n	%	n	%	n	%	n	%	n	%	n	%	n	%	n	%	n	%	
Patient number	10,140		9772		10,773		10,089		9058		8596		8183		7898		7248		6487		
Age																					
< 20	1176	11.60	880	9.01	767	7.12	518	5.13	387	4.27	266	3.09	202	2.47	153	1.94	95	1.31	68	1.05	
20–29	1115	11.00	1105	11.31	1183	10.98	1032	10.23	893	9.86	823	9.57	760	9.29	763	9.66	701	9.67	570	8.79	
30–39	1495	14.74	1586	16.23	1677	15.57	1655	16.40	1493	16.48	1532	17.82	1424	17.40	1471	18.63	1245	17.18	1057	16.29	
40–49	1486	14.65	1425	14.58	1554	14.43	1454	14.41	1372	15.15	1224	14.24	1174	14.35	1229	15.56	1127	15.55	1062	16.37	
50–59	1723	16.99	1686	17.25	1982	18.40	1837	18.21	1748	19.30	1607	18.69	1497	18.29	1445	18.30	1306	18.02	1161	17.90	
≥ 60	3145	31.02	3090	31.62	3610	33.51	3593	35.61	3165	34.94	3144	36.58	3126	38.20	2837	35.92	2774	38.27	2569	39.60	
Age (mean ± SD)	47.59	± 21.13	48.46	± 20.46	49.69	± 19.82	51.06	± 19.43	51.33	± 18.97	51.94	± 18.83	52.66	± 18.56	51.68	± 17.94	52.71	± 17.94	53.41	± 17.67	
Gender																					
Female	5612	55.35	5360	54.85	6037	56.04	5718	56.68	4984	55.02	4853	56.46	4577	55.93	4577	57.95	4156	57.34	3757	57.92	
Male	4528	44.65	4412	45.15	4736	43.96	4371	43.32	4074	44.98	3743	43.54	3606	44.07	3321	42.05	3092	42.66	2730	42.08	
Comorbidities																					
Diabetes mellitus	1711	16.87	1636	16.74	2068	19.20	2010	19.92	1772	19.56	1804	20.99	1750	21.39	1618	20.49	1493	20.60	1306	20.13	
Hypertension	3505	34.57	3449	35.29	4028	37.39	3977	39.42	3555	39.25	3408	39.65	3276	40.03	2966	37.55	2829	39.03	2511	38.71	
Allergic rhinitis	4347	42.87	4354	44.56	4792	44.48	4765	47.23	4340	47.91	4125	47.99	4009	48.99	3390	42.92	2984	41.17	2687	41.42	
Eczema	5383	53.09	5293	54.17	6073	56.37	5779	57.28	5315	58.68	5035	58.57	4813	58.82	4636	58.70	4217	58.18	3764	58.02	
Atopic dermatitis	678	6.69	692	7.08	770	7.15	802	7.95	682	7.53	631	7.34	637	7.78	585	7.41	528	7.28	463	7.14	
Dyslipidemia	2747	27.09	2712	27.75	3312	30.74	3315	32.86	3036	33.52	2979	34.66	2979	36.40	2829	35.82	2703	37.29	2454	37.83	
Coronary artery disease	1957	19.30	1987	20.33	2249	20.88	2191	21.72	2009	22.18	1785	20.77	1720	21.02	1570	19.88	1535	21.18	1348	20.78	
Congestive heart failure	460	4.54	434	4.44	504	4.68	508	5.04	472	5.21	407	4.73	444	5.43	366	4.63	357	4.93	326	5.03	
Chronic kidney disease	261	2.57	221	2.26	350	3.25	327	3.24	334	3.69	344	4.00	419	5.12	349	4.42	343	4.73	364	5.61	
Chronic hepatitis	460	4.54	434	4.44	504	4.68	508	5.04	472	5.21	407	4.73	444	5.43	366	4.63	357	4.93	326	5.03	
Stroke	1214	11.97	1215	12.43	1351	12.54	1368	13.56	1246	13.76	1141	13.27	1090	13.32	950	12.03	920	12.69	802	12.36	
Cancer	293	2.89	326	3.34	339	3.15	371	3.68	374	4.13	351	4.08	394	4.81	327	4.14	350	4.83	324	4.99	

Table 2 presents the seven common asthma medications, including oral steroids, intravenous steroids, SABA, ICS, ICS/ LABA, LAMA, and LTRA. Despite the yearly decrease in the number of asthma diagnoses, about 90% were treated with oral steroids, peaking at 97.29% in 2018. ICS usage also remained high, ranging from 86.20 to 91.75%. Additionally, the use of intravenous steroids increased from 40.94% in 2009 to 54.14% in 2018.

**Table 2:** The prescription pattern in patients with asthma by year

	2009		2010		2011		2012		2013		2014		2015		2016		2017		2018	
Variables	n	%	n	%	n	%	n	%	n	%	n	%	n	%	n	%	n	%	n	%
Oral steroids	9263	91.35	9022	92.33	10,078	93.55	9535	94.51	8578	94.70	8206	95.46	7808	95.42	7596	96.18	6993	96.48	6311	97.29
Intravenous steroids	4151	40.94	4270	43.70	4888	45.37	4819	47.76	4355	48.08	4290	49.91	4126	50.42	4004	50.70	3815	52.64	3512	54.14
Short-acting beta-agonists	1147	11.31	1090	11.15	1186	11.01	1168	11.58	1034	11.42	1019	11.85	978	11.95	896	11.34	809	11.16	746	11.50
Inhaled corticosteroids	8741	86.20	8457	86.54	9516	88.33	9046	89.66	8127	89.72	7736	90.00	7413	90.59	7193	91.07	6663	91.93	5952	91.75
Inhaled corticosteroids / long-acting beta agonists	238	2.35	254	2.60	321	2.98	275	2.73	278	3.07	275	3.20	274	3.35	222	2.81	187	2.58	168	2.59
Long-acting muscarinic antagonists	81	0.80	101	1.03	124	1.15	145	1.44	134	1.48	144	1.68	135	1.65	96	1.22	97	1.34	121	1.87
Leukotriene receptor antagonists	122	1.20	136	1.39	130	1.21	145	1.44	167	1.84	178	2.07	182	2.22	188	2.38	188	2.59	197	3.04

**Fig. 2:**
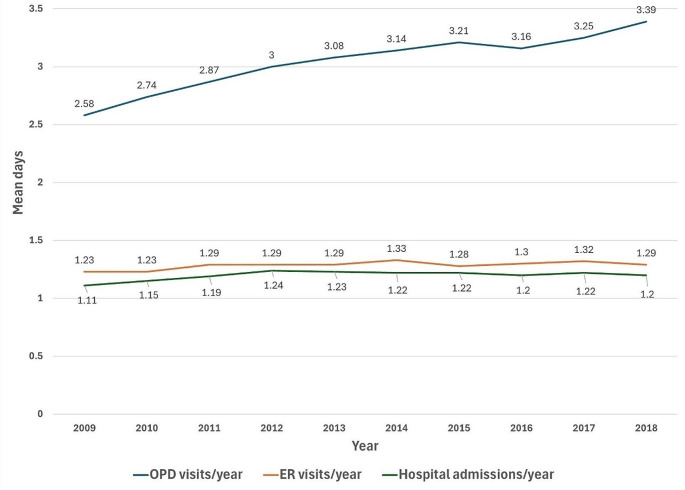
Trend line of the average number of OPD visits, ER visits, and hospitalizations for asthma patients per year from 2009 to 2018. ER: emergency room, OPD: outpatient department

**Fig. 3:**
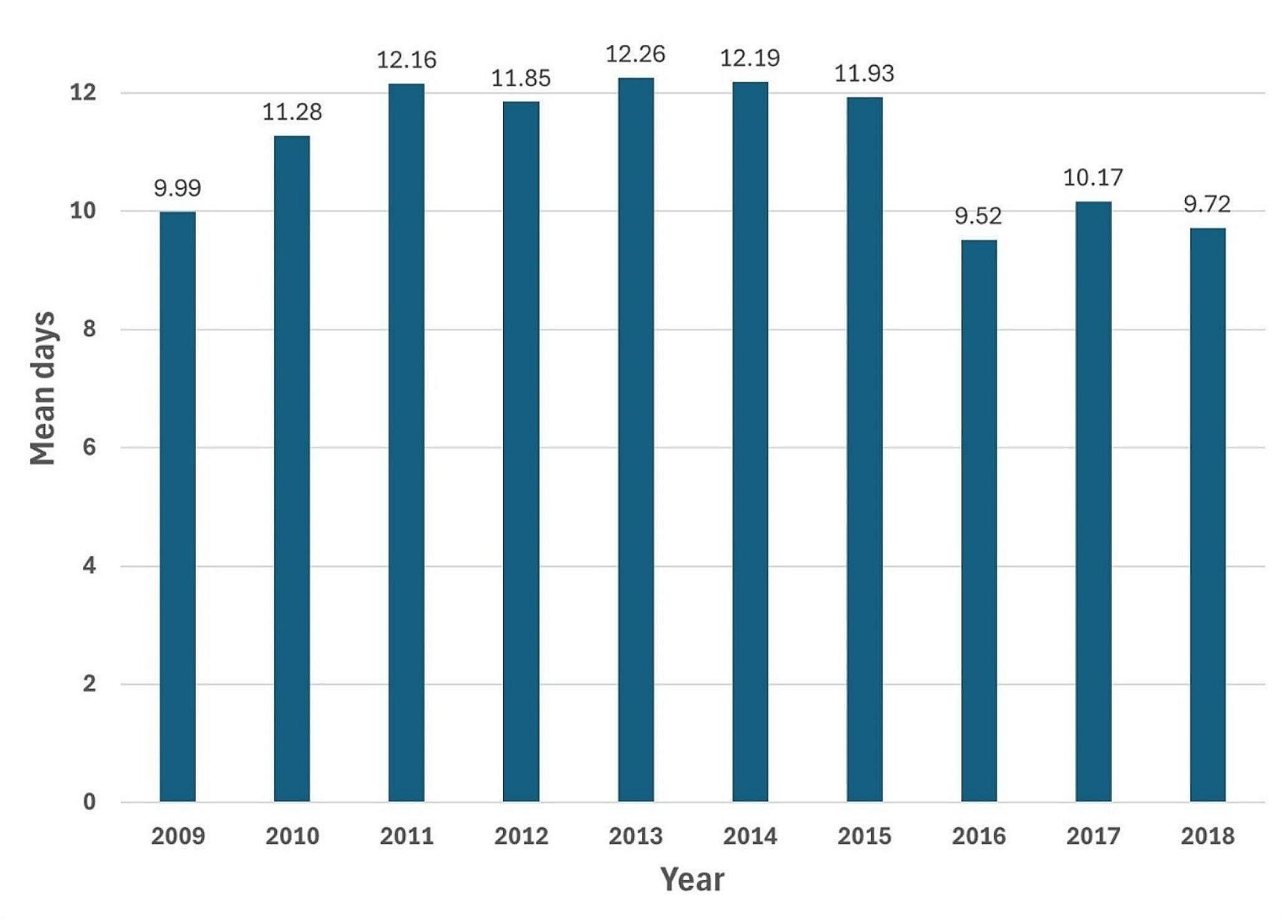
Trend line of the average days of hospitalization for asthma patients per year from 2009 to 2018

Table 3 shows the average number of OPD visits, ER visits, and hospitalizations, as well as the average days of hospitalization and medical costs for asthma patients per year over the past decade. Figure 2 shows the trend of OPD visits, ER visits, and hospitalizations in patients with asthma by years. From 2009 to 2018, the average annual OPD visits were approximately 2–3, while ER visits and hospitalizations were about 1. Figure 3 shows the average days that patients spend in hospital per year. The average length of stay in hospital varied from 9 to 12 days, reaching a maximum of 12.26 days (SD = 16.80) in 2013. Lastly, the average medical expenses per year ranged from NTD 5558 to 7922.

**Table 3:** The medical expenditure in patients with asthma by year

	2009		2010		2011		2012		2013		2014		2015		2016		2017			2018	
Variables	mean	SD	mean	SD	mean	SD	mean	SD	mean	SD	mean	SD	mean	SD	mean	SD	mean	SD	mean		SD
OPD visits/ year	**2.58**	**2.34**	**2.74**	**2.79**	**2.87**	**2.96**	**3.00**	**3.19**	**3.08**	**3.35**	**3.14**	**3.39**	**3.21**	**3.46**	**3.16**	**3.33**	**3.25**	**3.33**	**3.39**		**3.46**
ER visits/ year	**1.23**	**0.66**	**1.23**	**0.73**	**1.29**	**1.25**	**1.29**	**1.13**	**1.29**	**1.08**	**1.33**	**1.22**	**1.28**	**0.87**	**1.30**	**1.01**	**1.32**	**1.00**	**1.29**		**0.95**
Hospital admissions/ year	**1.11**	**0.41**	**1.15**	**0.51**	**1.19**	**0.69**	**1.24**	**0.86**	**1.23**	**0.87**	**1.22**	**0.73**	**1.22**	**0.72**	**1.20**	**0.64**	**1.22**	**0.71**	**1.20**		**0.69**
Average days of hospitalization/ year	**9.99**	**12.26**	**11.28**	**14.82**	**12.16**	**17.15**	**11.85**	**16.15**	**12.26**	**16.80**	**12.19**	**16.13**	**11.93**	**15.66**	**9.52**	**10.96**	**10.17**	**13.53**	**9.72**		**12.81**
Medical costs/ year (NTD)	**5558**	**26487.29**	**5641**	**23661.94**	**5862**	**27685.61**	**6145**	**27896.24**	**6576**	**28373.10**	**6616**	**26283.85**	**7077**	**29163.29**	**6314**	**23906.10**	**7324**	**38253.28**	**7922**		**42086.43**

ER: emergency room; NTD, new Taiwan dollar, 1 USD is equal to 33 NTD; OPD: outpatient department

Table 4 presents the characteristics of asthma patients with more than two hospitalizations per year. Since 2011, the number of patients has consistently exceeded 100, peaking at 171 in 2015, with over half being female. This group of patients with asthma experienced more than two hospitalizations (acute exacerbations) per year, indicating they may have a more severe form of asthma (difficult to control with the current treatment). The overall number of OPD visits, ER visits, and hospitalizations, as well as the length of hospital stay and medical costs, have increased.

**Table 4:** The medical expenditure in patients with asthma having multiple annual hospitalizations by year

	2009		2010		2011		2012		2013		2014			2015			2016			2017			2018		
Variables	n	%	n	%	n	%	n	%	n	%	n	%	n		%	n		%	n		%	n		%	
Patient number	52		78		102		140		139		155			171			143			160			166		
Age	62.12	± 20.38	65.88	± 19.97	63.15	± 21.27	67.01	± 18.80	63.45	± 21.52	63.12	± 21.76	64.20		± 19.86	62.16		± 18.36	60.16		± 20.43	58.14		± 21.24	
Gender																									
Female	31	51.62	40	51.28	53	51.96	77	55.00	70	50.36	84	54.19	103		60.23	102		71.33	108		67.50	114		68.67	
Male	21	40.38	38	48.72	49	48.04	63	45.00	69	49.64	71	45.81	68		39.77	41		28.67	52		32.50	52		31.33	
OPD visits/ year	7.03	± 6.65	5.41	± 3.77	7.66	± 7.18	7.33	± 5.77	6.40	± 5.51	6.94	± 5.99	7.42		± 5.81	7.61		± 6.37	7.19		± 6.27	8.08		± 6.47	
ER visits/ year	1.93	± 1.21	1.87	± 1.39	2.93	± 5.83	1.82	± 1.17	2.00	± 2.57	2.00	± 1.50	1.61		± 1.18	2.38		± 2.60	2.40		± 2.17	2.10		± 1.97	
Hospital admissions/ year	2.31	± 0.64	2.44	± 0.82	2.63	± 1.30	2.74	± 1.62	2.75	± 1.74	2.52	± 1.30	2.57		± 1.23	2.57		± 1.08	2.57		± 1.20	2.52		± 1.26	
Average days of hospitalization/ year	25.25	± 22.77	26.86	± 25.68	29.67	± 31.77	29.52	± 29.05	31.12	± 29.30	27.69	± 25.99	25.01		± 22.38	21.20		± 17.88	22.28		± 20.05	18.94		± 17.87	
Medical costs/ year (NTD)	126,109	± 110,567	124,323	± 114,640	145,732	± 155,704	147,140	± 132,257	151,994	± 144,193	140,156	± 125,500	130,503		± 116,627	123,303		± 97,162	142,051		± 131,061	140,984		± 199,686	

Age, OPD visits, ER visits, hospital admissions, average length of stay, and medical cost were shown as mean ± SD.

ER: emergency room; NTD, new Taiwan dollar, 1 USD is equal to 33 NTD; OPD: outpatient department

Table 5 details the dosages of asthma prescriptions for SABA, ICS, ICS/ LABA, and LAMA. The dosages of SABA < 75 mg comprised about 72%, while over 90% of the ICS dosages were < 3750 µg. The dosages of ICS/ LABA < 6304, 6304–16,500, > 16,500 µg were evenly distributed. From 2009 to 2013, most LAMA dosages exceeded 583,200 µg, but after 2014, dosages of < 4,500 µg accounted for about half.

**Table 5:** The prescription dose in patients with asthma by year

	2009		2010		2011		2012		2013		2014			2015			2016			2017			2018		
Variables	n	%	n	%	n	%	n	%	n	%	n	%	n		%	n		%	n		%	n		%	
Short-acting beta-agonists (mg)																									
< 5	518	45.16	423	38.81	484	40.81	463	39.64	417	40.33	408	40.04	369		37.73	328		36.61	305		37.70	289		38.74	
5–75	418	36.44	459	42.11	464	39.12	501	42.89	391	37.81	370	36.31	381		38.96	353		39.40	298		36.84	247		33.11	
> 75	211	18.40	208	19.08	238	20.07	204	17.47	226	21.86	241	23.65	228		23.31	215		24.00	206		25.46	210		28.15	
Inhaled corticosteroids (µg)																									
< 3750	8647	98.92	8378	99.07	9424	99.03	8976	99.23	8061	99.19	7676	99.22	7351		99.16	7142		99.29	6614		99.26	5896		99.06	
3750–40,000	41	0.47	28	0.33	37	0.39	26	0.29	27	0.33	34	0.44	32		0.43	31		0.43	28		0.42	30		0.50	
> 40,000	53	0.61	51	0.60	55	0.58	44	0.49	39	0.48	26	0.34	30		0.40	20		0.28	21		0.32	26		0.44	
Inhaled corticosteroids / long-acting beta agonists (µg)																									
< 6304	72	30.25	83	32.68	86	26.79	86	31.27	78	28.06	96	34.91	70		25.55	69		31.08	51		27.27	43		25.60	
6304–16,500	79	33.19	90	35.43	116	36.14	97	35.27	94	33.81	78	28.36	101		36.86	74		33.33	64		34.22	69		41.07	
> 16,500	87	36.55	81	31.89	119	37.07	92	33.45	106	38.13	101	36.73	103		37.59	79		35.59	72		38.50	56		33.33	
Long-acting muscarinic antagonists (µg)																									
< 4500	2	2.47	3	2.97	7	5.65	26	17.93	43	32.09	73	50.69	63		46.67	46		47.92	55		56.70	66		54.55	
4500-583,200	26	32.10	23	22.78	26	20.97	22	15.17	27	20.15	30	20.83	44		32.59	29		30.21	25		25.77	34		28.10	
> 583,200	53	65.43	75	74.26	91	73.39	97	66.90	64	47.76	41	28.47	28		20.74	21		21.88	17		17.53	21		17.36	

Table 6 shows the all-cause mortality rate of asthma per year. The mortality was calculated by dividing the total number of all-cause deaths of patients with asthma in a year by the total population in the same mid-year. All-cause mortality rates for the total asthma population, as well as for males and females, decreased annually. All-cause mortality rates of total asthma population, males, and females per 1000 were 0.46, 0.51, and 0.41 during 2009–2018, respectively.

**Table 6:** The mortality rate of asthma by years

	Overall			Male			Female			
Year	N	Mid-year population	Rate (per 10^3^ person)	n	Mid-year population	Rate (per 10^3^ person)	n	Mid-year population	Rate (per 10^3^ person)	
**2009**	1516	1,893,391	0.80	844	956,644	0.88	672	936,747	0.72	
**2010**	1316	1,871,081	0.70	761	944,554	0.81	555	926,527	0.60	
**2011**	1346	1,848,415	0.73	765	932,146	0.82	581	916,269	0.63	
**2012**	1167	1,824,765	0.64	650	919,403	0.71	517	905,362	0.57	
**2013**	901	1,801,870	0.50	509	907,124	0.56	392	894,746	0.44	
**2014**	719	1,779,335	0.41	387	894,895	0.43	332	884,440	0.38	
**2015**	643	1,757,286	0.37	355	883,013	0.40	288	874,273	0.33	
**2016**	337	1,735,147	0.19	167	871,060	0.19	170	864,087	0.20	
**2017**	227	1,713,217	0.13	106	859,364	0.12	121	853,853	0.14	
**2018**	108	1,692,070	0.06	52	847,987	0.06	56	844,083	0.07	
**Mean**			0.46			0.51			0.41	

Table 7 shows the incidence rate of asthma during the study period. The incidence rates for total asthma population, as well as males and females, also decreased annually. The incidence rates per 1000 were 4.93 for overall, 4.28 for males, and 5.58 for females during 2009–2018, respectively.

**Table 7:** The incidence rate of asthma by years

	Overall			Male			Female			
**Year**	**n**	**Mid-year population**	**Rate (per 10** ^**3**^ **person)**	**n**	**Mid-year population**	**Rate (per 10** ^**3**^ **person)**	**n**	**Mid-year population**	**Rate (per 10** ^**3**^ **person)**	
**2009**	10,140	1,893,391	5.36	4528	956,644	4.73	5612	936,747	5.99	
**2010**	9772	1,871,081	5.22	4412	944,554	4.67	5360	926,527	5.79	
**2011**	10,773	1,848,415	5.83	4736	932,146	5.08	6037	916,269	6.59	
**2012**	10,089	1,824,765	5.53	4371	919,403	4.75	5718	905,362	6.32	
**2013**	9058	1,801,870	5.03	4074	907,124	4.49	4984	894,746	5.57	
**2014**	8596	1,779,335	4.83	3743	894,895	4.18	4853	884,440	5.49	
**2015**	8183	1,757,286	4.66	3606	883,013	4.08	4577	874,273	5.24	
**2016**	7898	1,735,147	4.55	3321	871,060	3.81	4577	864,087	5.30	
**2017**	7248	1,713,217	4.23	3092	859,364	3.60	4153	853,853	4.87	
**2018**	6487	1,692,070	3.83	2730	847,987	3.22	3757	844,083	4.45	
**Mean**			4.93			4.28			5.58	

## Discussion

Our data indicates a declining trend in asthma incidence rates from 2009 to 2018 for overall, male, and female populations. The total incidence rates of asthma per 1000 persons for overall, male, and female are 4.93, 4.28, and 5.58, respectively. Additionally, the data reveals that the average annual OPD visits ranged from 2 to 3, with ER visits and hospitalizations typically around 1. The duration of hospital stays fluctuated between 9 and 12 days per year. Furthermore, the annual mortality rates for the overall, male, and female populations have been decreasing. The overall mortality rates per 1000 asthma patients were 0.46 for the total population, 0.51 for males, and 0.41 for females.

Many studies provide substantial evidence of a connection between allergic rhinitis and asthma, consistently showing they often occur together. Findings reveal that allergic rhinitis are present in 28–78% of patients with asthma [12–14], markedly more than the 5–20% rate found in the general population [15]. A guideline report that as many as 38% of people with allergic rhinitis are also affected by asthma, while between 6% and 85% of those with asthma report experiencing nasal symptoms [16]. In our data, we conducted a 10-year follow-up and found that 42–49% of patients with asthma also had allergic rhinitis, which is consistent with findings from previous studies. The presence of allergic rhinitis significantly impacts severe asthma, elevating its incidence among both older and younger populations. This link between allergic rhinitis and heightened asthma severity has been documented in studies involving both children and adults, as noted by several researchers [17–20].

Atopic dermatitis and eczema, persistent inflammatory skin conditions often linked with respiratory allergies, stand as prevalent skin disorders seen in those with asthma. Asthma, eczema, and atopic dermatitis share numerous comorbidities and are associated with one another, potentially due to immunological disruption, especially the expression of Th2 cytokines [21]. The prevalence of eczema in asthma patients varies, indicating a significant overlap between eczema and other allergic conditions, including asthma. Atopic dermatitis, the most common form of eczema, is strongly linked to a personal or family history of asthma and other allergic diseases, suggesting a high rate of co-occurrence. Previous studies, including one by Pourpak et al. [22], indicate that asthma occurs more frequently in patients with atopic dermatitis, though the exact causative factors are unclear. According to Pourpak et al. [22], 27.5% of patients with atopic dermatitis were found to have asthma. However, there is limited information regarding the prevalence of eczema and atopic dermatitis in patients with asthma. Nonetheless, our data highlight a significant correlation between eczema and asthma, particularly among individuals with asthma and the association between eczema and varying degrees of asthma severity warrants further investigation.

From our data, we found that over 90% of patients with asthma had at some point received oral steroids, and more than 50% had received intravascular steroids after 2015. The trend for both oral and intravascular steroids use has been increasing. This may suggest that many asthma patients have poor control or frequent acute exacerbations, necessitating systemic steroids treatment. Despite a 2010 study showing that adding LAMA to inhaled corticosteroids can improve symptoms and lung function in patients with inadequately controlled asthma, the percentage of asthma patients receiving LAMA treatment remains low.

The provision to exempt extended prescriptions for chronic diseases after a visit to the outpatient department was designed to reduce unnecessary outpatient visits for medication refills in Taiwan. Patients with stable chronic conditions can utilize this exemption to receive free medications for up to 60 days after obtaining a 30-day supply from the hospital. After receiving this initial supply from the hospital’s outpatient department, most patients can directly acquire an additional 60 days of outpatient prescription drugs from pharmacy stores. Patients with good adherence to their chronic disease management typically need to visit the hospital four times a year. According to our data, the average number of outpatient department visits for patients with asthma was 3.39 in 2018. This suggests that there is room for improvement in asthma treatment adherence.

Observational and database research has shown that patients with asthma who have a history of frequent exacerbations or eosinophilic inflammation face an increased risk of subsequent acute exacerbation [23–25].

Patients identified in respective US and UK databases were found to have varying experiences based on their asthma severity. Those with severe asthma, a history of two or more exacerbations compared to fewer than two, and severe uncontrolled asthma versus non-severe asthma experienced the highest rates of asthma-related emergency department visits and hospital readmissions within 30 days [26]. We analyzed our patients with asthma who had more than two hospitalizations per year. Since 2011, the number of patients in this group has consistently exceeded 100, reaching a peak of 171 in 2015, with over half being female. Their mean age ranged from 58 to 67, and they needed to OPD visits an average of 8 times per year, with an average of 2 ER visits annually. The length of their hospital stays ranged from 19 to 31 days per year. This group incurred significantly higher medical costs compared to the general asthma population.

Drawing from the 2019 Global Burden of Disease study, research outlined spatial and temporal patterns in asthma incidence and mortality worldwide and in 204 countries from 1990 to 2019. Over this period, asthma incidence declined from 6.01 per 1,000 to 4.78 per 1,000, while asthma mortality rates fell from 8.60 per 100,000 to 5.96 per 100,000 [27]. Our data show comparable trends. Between 2009 and 2018, the incidence rates and mortality in patients with asthma for the overall population, as well as for males and females, have consistently decreased each year. Specifically, the total incidence rates for the overall population, males, and females are 4.93, 4.28, and 5.58 per 1,000 persons, respectively.

There was a widespread trend of observing lower age-standardized asthma mortality rates in developed countries, while undeveloped countries exhibited higher mortality rates. This difference implies that unequal access to preventive and treatment services among socioeconomically disadvantaged groups could impede continuous advancements in reducing asthma-related deaths. In numerous low-income and lower-middle-income countries, issues like underdiagnosis, misdiagnosis, and insufficient treatment lead to many preventable asthma incidents and fatalities [28].

The study encountered several limitations. One primary issue was the reliance on physician diagnoses for asthma without a universally accepted physiological benchmark. The precision of asthma incidence measurements was influenced by access to healthcare. Furthermore, the analysis of incidence was based on estimated cross-sectional data from the population at mid-year. On the positive side, a major strength of this study lies in its national scale, employing a comprehensive insurance database that covers more than 99% of the population in Taiwan. This broad reach provides a strong foundation for the credible observation of a decreasing asthma incidence and mortality trend in Taiwan.

## Conclusion

In summary, both asthma incidence and mortality rates decreased in Taiwan from 2009 to 2018. There is potential to improve adherence among asthma patients. The utilization of LAMA can be increased in patients with asthma who continue to experience symptoms and poor control despite receiving ICS/ LABA treatment. Patients with asthma who are hospitalized twice a year incur higher medical costs compared to the general asthma population.

## Data Availability

No datasets were generated or analysed during the current study.

## Abbreviations

ATC: Anatomical therapeutic chemical
ER: Emergency room
FF: Fluticasone furoate
GAN: Global Asthma Network
ICD-9-CM: International Classification of Diseases, 9th Revision, Clinical Modification
ICD-10-CM: International Classification of Diseases, 10th Revision, Clinical Modification
ICS: Inhaled corticosteroids
ISAAC: International Study of Asthma and Allergies in Childhood
NHI: National Health Insurance
OPD: Outpatient department
SABA: Short-acting beta-agonists
LABA: Long-acting beta-agonists
LAMA: Long-acting muscarinic antagonists
LTRA: Leukotriene receptor antagonist
VI: Vilanterol
